# 1,8-Cineol Reduces Mucus-Production in a Novel Human *Ex Vivo* Model of Late Rhinosinusitis

**DOI:** 10.1371/journal.pone.0133040

**Published:** 2015-07-24

**Authors:** Holger Sudhoff, Christin Klenke, Johannes F. W. Greiner, Janine Müller, Viktoria Brotzmann, Jörg Ebmeyer, Barbara Kaltschmidt, Christian Kaltschmidt

**Affiliations:** 1 Department of Otolaryngology, Head and Neck Surgery, Klinikum Bielefeld, D-33604 Bielefeld, Germany; 2 Department of Cell Biology, University of Bielefeld, D-33501 Bielefeld, Germany; 3 AG Molecular Neurobiology, University of Bielefeld, D-33501 Bielefeld, Germany; University of Pittsburgh, UNITED STATES

## Abstract

Inflammatory diseases of the respiratory system such as rhinosinusitis, chronic obstructive pulmonary disease, or bronchial asthma are strongly associated with overproduction and hypersecretion of mucus lining the epithelial airway surface. 1,8-cineol, the active ingredient of the pharmaceutical drug Soledum, is commonly applied for treating such inflammatory airway diseases. However, its potential effects on mucus overproduction still remain unclear.In the present study, we successfully established *ex vivo* cultures of human nasal turbinate slices to investigate the effects of 1,8-cineol on mucus hypersecretion in experimentally induced rhinosinusitis. The presence of acetyl-α-tubulin-positive cilia confirmed the integrity of the *ex vivo* cultured epithelium. Mucin-filled goblet cells were also detectable in nasal slice cultures, as revealed by Alcian Blue and Periodic acid-Schiff stainings. Treatment of nasal slice cultures with lipopolysaccharides mimicking bacterial infection as observed during late rhinosinusitis led to a significantly increased number of mucin-filled goblet cells. Notably, the number of mucin-filled goblet cells was found to be significantly decreased after co-treatment with 1,8-cineol. On a molecular level, real time PCR-analysis further showed 1,8-cineol to significantly reduce the expression levels of the mucin genes MUC2 and MUC19 in close association with significantly attenuated NF-κB-activity. In conclusion, we demonstrate for the first time a 1,8-cineol-dependent reduction of mucin-filled goblet cells and MUC2-gene expression associated with an attenuated NF-κB-activity in human nasal slice cultures. Our findings suggest that these effects partially account for the clinical benefits of 1,8-cineol-based therapy during rhinosinusitis. Therefore, topical application of 1,8-cineol may offer a novel therapeutic approach to reduce bacteria-induced mucus hypersecretion.

## Introduction

The clearance of mucus represents a primary defense mechanism within mammalian airways, providing a protective barrier against pathogens and toxins [1]. Overproduction and hypersecretion of mucin are common symptoms of inflammatory diseases, such as rhinosinusitis, chronic obstructive pulmonary disease (COPD) [2, 3] or asthma [4]. As a mixture of water, ions, glycoproteins, and lipids, mucus coats the apical epithelial surface of the human respiratory tract. Mucosal components are secreted by goblet cells in polarized epithelium and by secretory cells in the submucosal glands (SMG) [5]. The major macromolecular constituents of epithelial mucus are mucins, which are large, highly glycosylated, viscoelastic macromolecules. Mucin glycoproteins are particularly involved in organizing the structure of the mucus and mainly contribute to its rheological properties [6, 7]. Likewise, mucin overproduction is strongly associated to inflammatory diseases, like rhinosinusitis [8], cystic fibrosis [9], chronic bronchitis [10] or asthma [11, 12].

Medical treatment of inflammatory diseases of the human respiratory tract may involve 1,8-cineol, the active ingredient of the clinically-accredited medical product Soledum. 1,8-cineol was identified by Cloez in the 1870s as the major constituent of *Eucalyptus globulus* essential oil [13] and possesses both anti-microbial [14] and anti-inflammatory properties [15, 16]. In 2005, Inoue and colleagues demonstrated 1,8-cineol to partially reduce airway inflammation in a mouse model of allergic asthma [17]. Accordingly, placebo-controlled double-blind trials impressively showed the beneficial anti-inflammatory activity of 1,8-cineol for treating inflammatory diseases, as in rhinosinusitis [18], bronchial asthma [19], and COPD [20]. Despite these promising findings, a direct linkage between the anti-inflammatory activity of 1,8-cineol and mucus production as a hallmark of inflammatory diseases remains undetermined.

Facing these challenges, we aimed to establish an *ex vivo* culture system for human nasal turbinate tissue to investigate the potential effects of 1,8-cineol on mucus hypersecretion during experimentally induced rhinosinusitis. Although mouse models of rhinosinusitis [21, 22], cultured primary human airway epithelial cells, or human bronchial explants [23] are commonly used to model and mimic inflammation, these models may not fully reflect the complex pathology of diseased human nasal tissue. In the present study, nasal epithelia were successfully cultivated *ex vivo* and retained their integrity, including the presence of mucin-filled goblet cells. Notably, treatment of nasal slice cultures with lipopolysaccharides (LPS) mimicking bacterial infection common during late rhinosinusitis was associated with a significantly increased number of mucin-filled cells. A co-treatment with 1,8-cineol led to a significantly attenuated density of this specific cell type. Accordingly, 1,8-cineol-treatment resulted in a significantly reduced expression of the mucin genes MUC2 and MUC19 levels in a close association with attenuated activity of NF-κB. Our findings suggest the role of 1,8-cineol in reducing mucus overproduction to contribute to the beneficial effects of 1,8-cineol-based therapeutic approaches.

## Material and Methods

### Human Material

Human nasal turbinate specimens (middle and inferior turbinates) were obtained from patients during minimal-invasive surgery after informed written consent and according to the principles of the Declaration of Helsinki (1964) and local and international guidelines (Bezirksregierung Detmold/Münster). Fifty percent of the patients were pre-treated with decongestant spray containing Xylometazolin (Nasic, pharmacological half-life of 12h, Klosterfrau Healthcare Group) prior to obtaining nasal inferior turbinate tissue. Isolation and further experimental procedures were ethically approved by the ethics commission of the Ärztekammer Westfalen-Lippe and the medical faculty of the Westfälische Wilhems-Universität (Münster, Germany) (approval reference number 2012-15-fS).

### Tissue culture

Specimens obtained during surgery were directly placed on ice in Dulbecco modified Eagle medium (PAA, Pasching, Austria) supplemented with penicillin and streptomycin. For tissue culture, specimens were sliced (200μm thickness) using McIlwain tissue chopper (Ted Pella, Inc., Redding, CA) before being transferred to culture plate inserts comprising a 0.4 μm nitrocellulose membrane (Millipore/Greiner, Billerica, MA, USA). Slice-containing membranes were placed at the interface of air and B-ALI differentiation medium (Lonza, Basel, Switzerland) within respective cell culture well-plates (TPP Techno Plastic Products, Trasadingen, Austria) and DMEM High Glucose (Biochrom, Berlin, Germany) followed by culture in a humidified incubator (Binder, Tuttlingen, Germany) at 37°C and 5% CO_2_. Nasal slices were cultured for up to 4 weeks and fed every two days by replacing the medium with fresh B-ALI differentiation medium.

Human nasal slices cultivated for 7 days were treated with LPS (100 ng/ml, rough strains from Salmonella enterica Re 595, cat. no. L9764, Sigma-Adrich St. Louis, MO, USA) or LPS and 1,8-Cineol (10^−5^ M, Klosterfrau Healthcare Group, Cassella-med GmbH & Co. KG, Cologne, Germany as described in [24] for 60 minutes followed by respective stainings or real time PCR.

### Immunohistochemistry

Nasal slice cultures were fixed using 4% formalin, treated with water for 60 minutes and dehydrated and embedded by applying 50% EtOH, 75% EtOH, 95% EtOH, 100% EtOH, Xylol and Xylol:Paraplast (1:1, Leica Biosystems Nussloch GmbH, Nussloch, Germany) for 60 minutes each followed by application of Paraplast over night. Afterwards, 2 μm thick sections were prepared. Prior to immunohistochemistry, sections were deparaffinized in Xylene and afterwards rehydrated in 100% EtOH for 3 minutes, 95% EtOH for 3 minutes and 70% EtOH for 3 minutes followed by application of distilled water.

Alternatively, 10 μm thick cryostat sections were prepared from nasal slices cultures and inferior turbinate tissue followed by fixation using 4% paraformaldehyde for 20 min at RT prior to immunohistochemistry.

Blocking was performed using 5% goat serum for 30 minutes followed by incubation with primary rabbit anti-acetyl-α-tubulin antibody (1:800, Cell Signaling Technology, Danvers, MA, USA), anti-Caspase 3 antibody (1:500, Cell Signaling) or anti-p65 antibody (Santa Cruz Biotechnology, Heidelberg, Germany) for 2 hours at RT. Secondary fluorochrome-conjugated antibody (goat anti rabbit conjugated with Alexa 555, 1:300; Molecular Probes, Göttingen, Germany) applied for 1 hour at room temperature (RT). Nuclear counterstaining was performed using SYTOX green (1:20000, Invitrogen, Carlsbad, CA, USA) for 30 minutes at RT. Fluorescence imaging was performed using confocal laser scanning microscopy (LSM 510, Carl Zeiss, and DM IRB, Leica, Wetzlar, Germany).

### Staining

Cultured nasal slices were embedded in paraffin and sectioned as described above. For Hematoxylin and Eosin (HE) staining sections were deparaffinized in Xylene and afterwards rehydrated in 100% EtOH for 3 minutes, 95% EtOH for 3 minutes and 70% EtOH for 3 minutes. After rinsing the sections in distilled water, Mayer’s alum haematoxylin was applied for 5 minutes and rinsed in running tap water. Afterwards, sections were stained for 2 minutes using 2% Eosin solution and immersed once in water. Sections were dehydrated by rinsing in 70% Ethanol for 3 minutes, 90% Ethanol for 3 minutes and two times 100% Ethanol for 3 minutes each. Finally, the slides were cleared using Xylene and mounted in Entellan.

For Alcian Blue staining sections were deparaffinized and rehydrated as described above. Rehydrated sections were stained for 8 minutes in Alcian blue solution (1g Alcian blue, 100 ml 3% acetic acid, pH 2.5) and rinsed in running tap water for 2 minutes. Afterwards, 0.1% Nuclear fast red solution (0.1g nuclear fast red, 5g Aluminum sulfate, 100ml distilled water) was applied for 10 minutes followed by rinsing in running water for 3 minutes. Sections were dehydrated, cleared and finally mounted in Entellan as described above.

### PAS (Periodic Acid Solution) Staining

Sections were deparaffinized and hydrated to water. 0.5% periodic acid solution was applied for 5 minutes. After rinsed in distilled water Schiff reagent was placed for 15 minutes. Washing in lukewarm tap water for 5 minutes was followed by a counterstaining in Mayer's hematoxylin for 1 minute. Again the sections were washed in tap water for 5 minutes, followed by dehydration and embedding using a synthetic mounting medium.

### Real time PCR

Total RNA was isolated from tissue cultures of three donors after respective LPS- or LPS and 1,8-cineol treatment using the MasterPure RNA Purification Kit (Biozym Scientific GmbH, Hessisch Oldendorf, Germany) followed by cDNA synthesis by First Strand cDNA Synthesis Kit (Thermofisher, St. Leon-Rot, Germany) according to the manufacturer’s guidelines. qPCR reactions were performed in technical triplicates using myBudget 5x EvaGreen QPCR-Mix II (ROX) (BioBudget Technologies GmbH, Krefeld, Germany) according to the manufacturer’s guidelines and assayed with a ABI PRISM 7000 Sequence detection system (Thermo Fisher Scientific, Bonn, Germany). For primer sequences see Table 1.

**Table 1:** Primer Sequences.

target	forward primer	reverse primer
MUC5AC	ACCTCTGCTCCTACAACCAGAACA	GAAGTCCACGTCGAACCACTTTGT
MUC5B	TATTCCACCTTTGACGGCACCTCT	CTGCTCACCGGAATTTGGTCAAAC
MUC19	TCCCTAGGTGGAAGTGCAATGACA	TCTGGATTCACTTCCGGTACTGCT
MUC2	AGCCCGGTTCTCCAGTTTATTCCT	ATGAGCTGGTTGTGGATCTTCACG
TNFα	AAGCCCTGGTATGAGCCCATCTAT	AGGGCAATGATCCCAAAGTAGACC

## Results

### Cultured human nasal slices reveal intact ciliated surface and mucus-filled goblet cells

**Fig 1:**
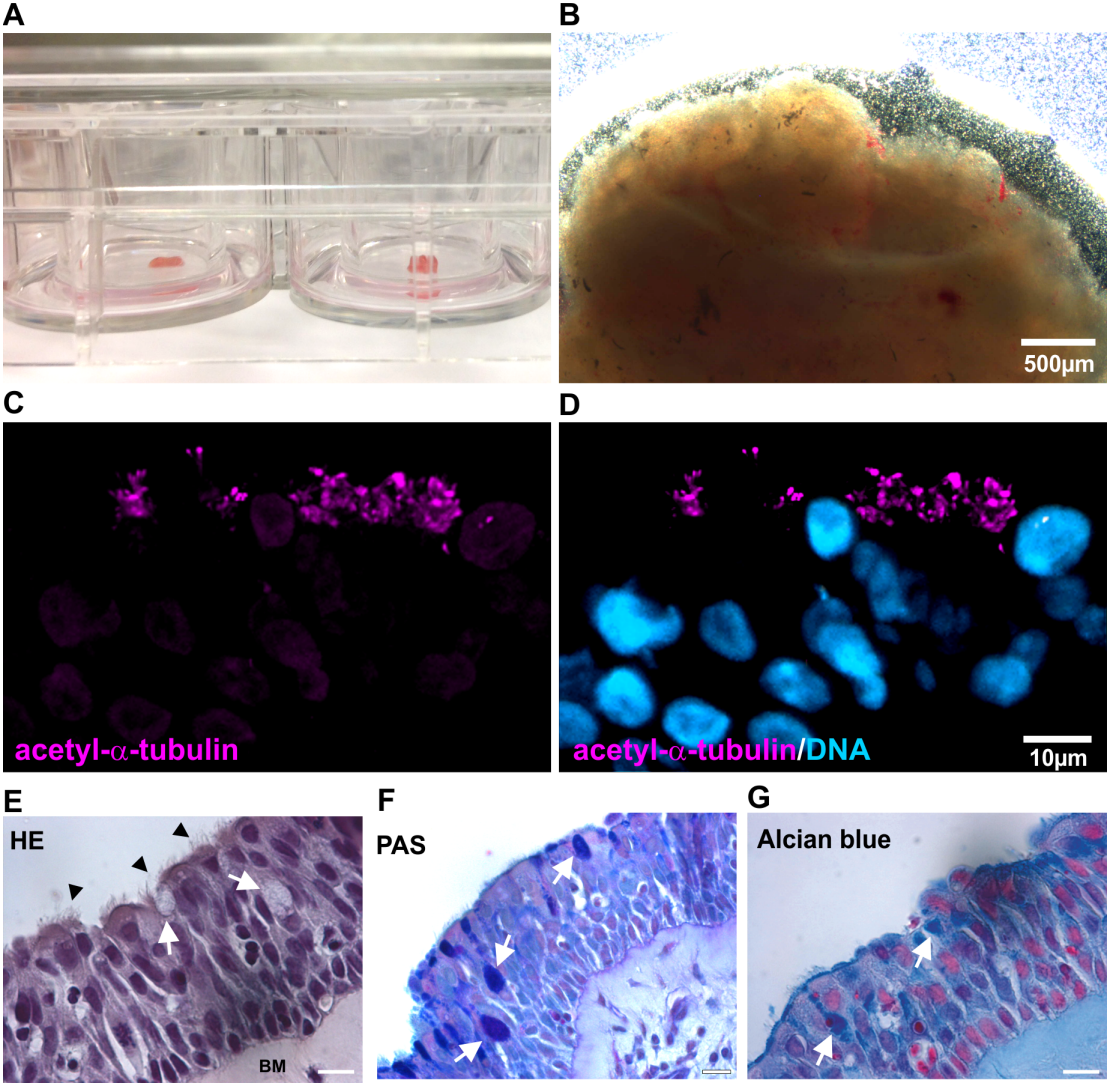
Cultured human nasal slices show unimpaired epithelium containing mucus-filled goblet cells. **A,B**: Overview images of the established nasal slice culture system showing sliced nasal tissue cultured in culture plate inserts within a 12-well-plate. **C,D**: Immunohistochemical staining revealed the presence of acetyl-α-tubulin-positive cilia in nasal slice cultures. **E**: Hematoxylin and eosin-staining displayed the integrity of the *ex vivo* cultured epithelium containing ciliated epithelial cells (arrowheads), goblet cells (arrows) and a basal membrane (BM). Scale Bar: 20 μm. **F, G**: Mucin-filled goblet cells (arrows) were detected in cultivated nasal slices by Alcian Blue-staining and Periodic acid-Schiff stain. Scale Bar: 20 μm.

To investigate potential effects of 1,8-cineol on mucus hypersecretion, we applied *ex vivo* cultures of sliced human nasal turbinate tissue (Fig 1A and 1B). Determining the integrity of the cultured nasal tissue, immunohistochemical stainings revealed the presence of acetyl-α-tubulin-positive cilia in nasal slices cultured for 32 days (Fig 1C and 1D). Hematoxylin and eosin-stained ciliated epithelial cells forming an intact ciliated surface (Fig 1E, arrowheads), goblet cells (Fig 1E, arrows) as well as the presence of a basal membrane (Fig 1E, BM) further confirmed the integrity of the *ex vivo* cultured epithelium [25, 26]. In addition, cultured nasal tissue showed no changes in Caspase 3-expression compared to inferior turbinate tissue, suggesting unaffected viability of the cultured nasal tissue (S1 Fig). The presence of goblet cells was further investigated using Alcian Blue-staining and Periodic acid-Schiff stain detecting mucins [27]. As depicted in Fig 1E–1G, mucin-filled goblet cells were visible in cultivated nasal slices (arrowheads), suggesting the applicability of the here established culture system to investigate potential effects of 1,8-cineol on mucus overproduction.

### Increased number of mucus-stuffed goblet cells in LPS-treated nasal slice cultures is significantly reduced by co-treatment with 1,8-cineol

**Fig 2:**
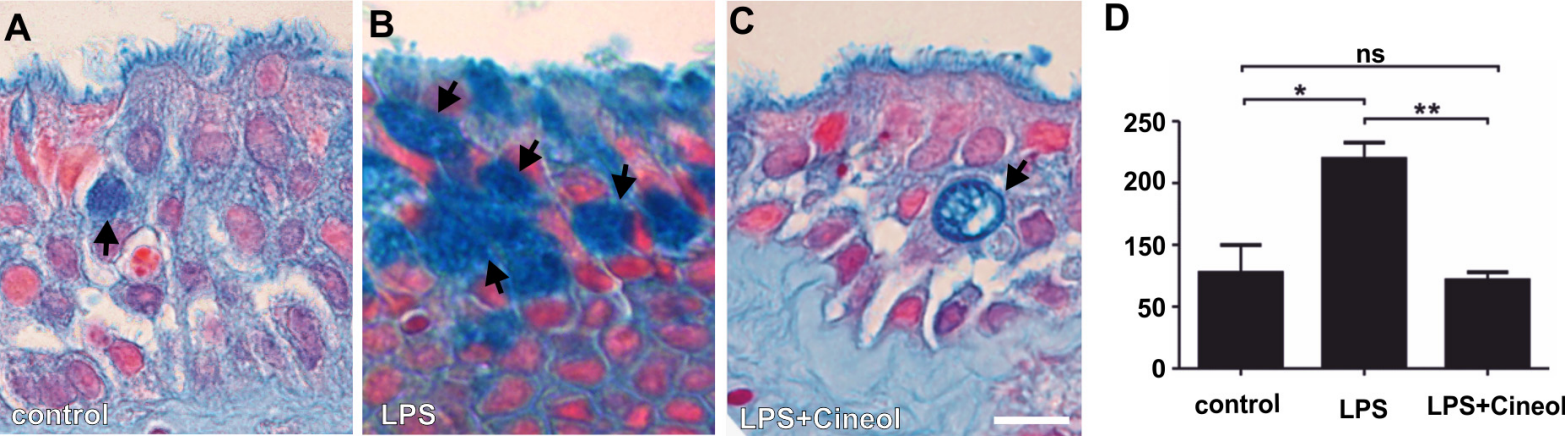
Increased number of mucus-filled cells in LPS-treated nasal slice cultures is significantly reduced by co-treatment with 1,8-cineol. **A**: Representative Alcian Blue-staining of an untreated nasal slice culture revealed no increased amount of mucus-filled goblet cells (arrows). **B**: Representative Alcian Blue-staining of LPS-treated nasal slices showed highly increased numbers of mucus-filled goblet cells (Arrows). **C**: Representative Alcian Blue-staining of cultured nasal slices co-treated with LPS and 1,8-cineol displayed a highly decreased number of mucus-filled goblet cells (Arrows). Scale Bar: 20 μm. **D**: Quantification of total areas of Alcian Blue-stained slice cultures from four independent donors revealed a significantly increased number of mucin-filled goblet cells in LPS-treated nasal slice cultures, which was significantly decreased after co-treatment with 1,8-cineol. *p < 0.5, **p < 0.01 were considered significant (t-test); ns: not significant (t-test).

Simulating the presence of a bacterial cell wall, we treated cultivated nasal slices with LPS to determine potential effects of 1,8-cineol on mucin overproduction during experimentally induced late rhinosinusitis [28]. Here, Alcian Blue-staining revealed a highly increased number of mucin-filled goblet cells in cultured tissue after LPS-treatment (Fig 2B, arrows) in contrast to the untreated control approach (Fig 2A, arrows). Notably, the number of mucin-filled goblet cells was decreased in cultured nasal slices treated with LPS and 1,8-cineol (Fig 2C, arrows). Determining these effects in more detail, quantification of mucus-filled goblet cells revealed a significantly increased number of mucin-filled cells after LPS-treatment, which was significantly decreased after co-treatment with 1,8-cineol (Fig 2D). In addition, cultured nasal slices co-treated with LPS and 1,8-cineol showed unchanged low numbers of Caspase 3-expressing cells compared to untreated and LPS-treated cultures as well as to inferior turbinate tissue, suggesting unaffected viability of the cultured nasal tissue (S1 Fig).

### Exposure of nasal slice cultures to 1,8-cineol leads to significantly decreased levels of MUC gene expression associated with reduced activity of NF-κB

**Fig 3:**
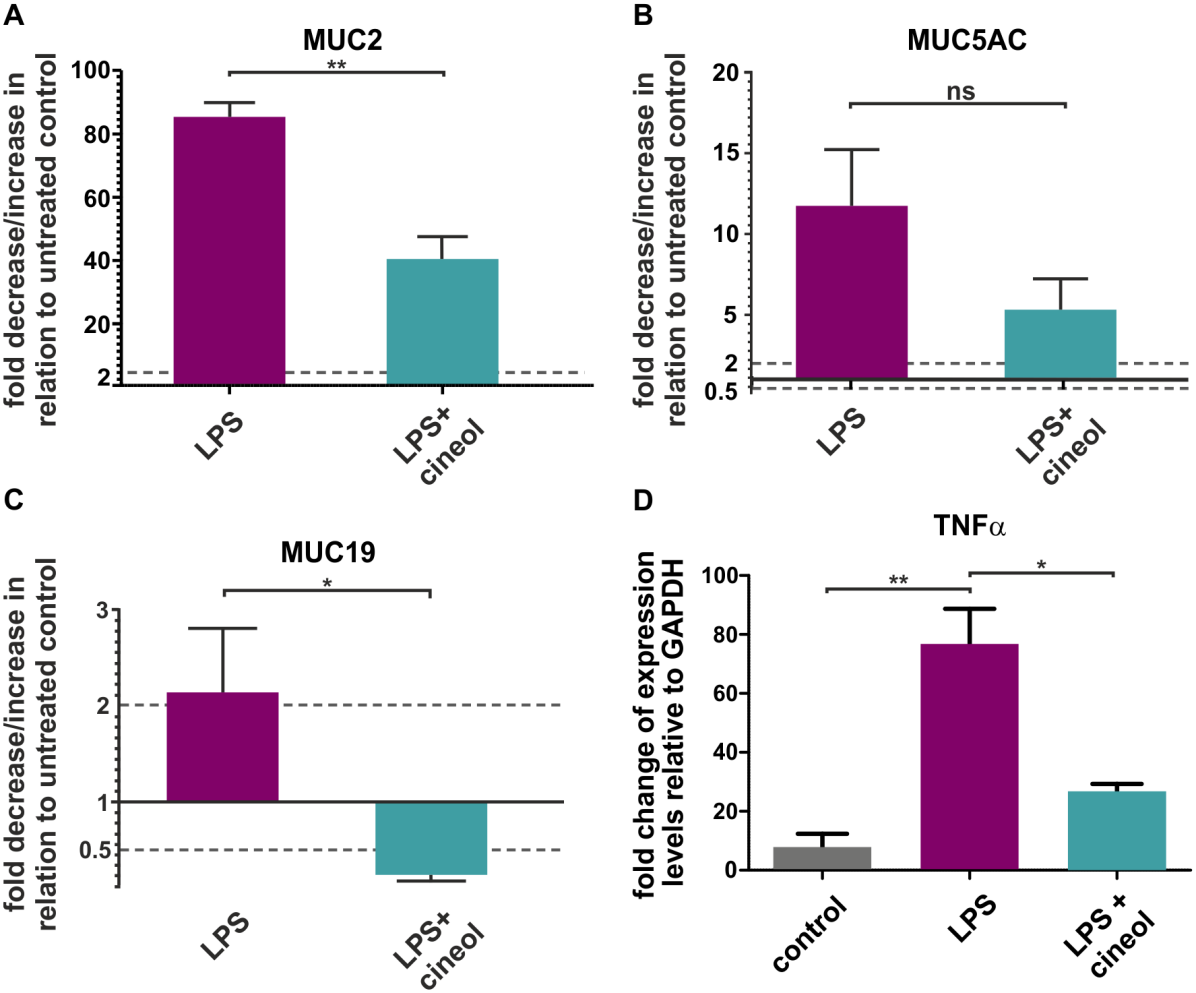
1,8-cineol-treamtent leads to significantly decreased levels of MUC gene expression after their LPS-dependent stimulation. **A**: Real time PCR analyses of nasal slice culture depicted increased levels of MUC2 after LPS-treatment, which were significantly reduced in LPS- and 1,8-cineol-treated approaches. **B**: No significant changes in gene expression level of MUC5AC in nasal slice cultures after LPS- as well as LPS- and 1,8-cineol-treatment shown by real time PCR. **C**: Real time PCR analyses revealed decreased levels of MUC19 in nasal slice cultures co-treated with LPS- and 1,8-cineol in comparison to LPS-treated approaches. **D**: Real time PCR analyses showed decreased expression levels of TNFα in nasal slice cultures co-treated with LPS- and 1,8-cineol compared to LPS-treated approaches. *p < 0.5, **p < 0.01 were considered significant (t-test); ns: not significant (t-test). GAPDH: Glyceraldehyde 3-phosphate dehydrogenase.

Investigating the effects of 1,8-cineol on mucus overproduction on a molecular level, we examined LPS as well as LPS- and 1,8-cineol-treated nasal slice cultures with real time PCR analysis to determine levels of mucin gene expression. Here, co-treatment of nasal tissue with LPS and 1,8-cineol was significantly reduce the expression level of the mucin gene MUC2 in comparison to LPS-stimulated samples (Fig 3A). However, the expression levels of the mucin genes MUC5AC and MUC5B were not significantly reduced in LPS-treated nasal slice cultures after co-treatment with 1,8-cineol (Fig 3B, data not shown). Interestingly, the expression level of MUC19 was also shown to be significantly reduced in slice cultures treated with LPS and 1,8-cineol in comparison to the LPS-treated approach (Fig 3C).

**Fig 4:**
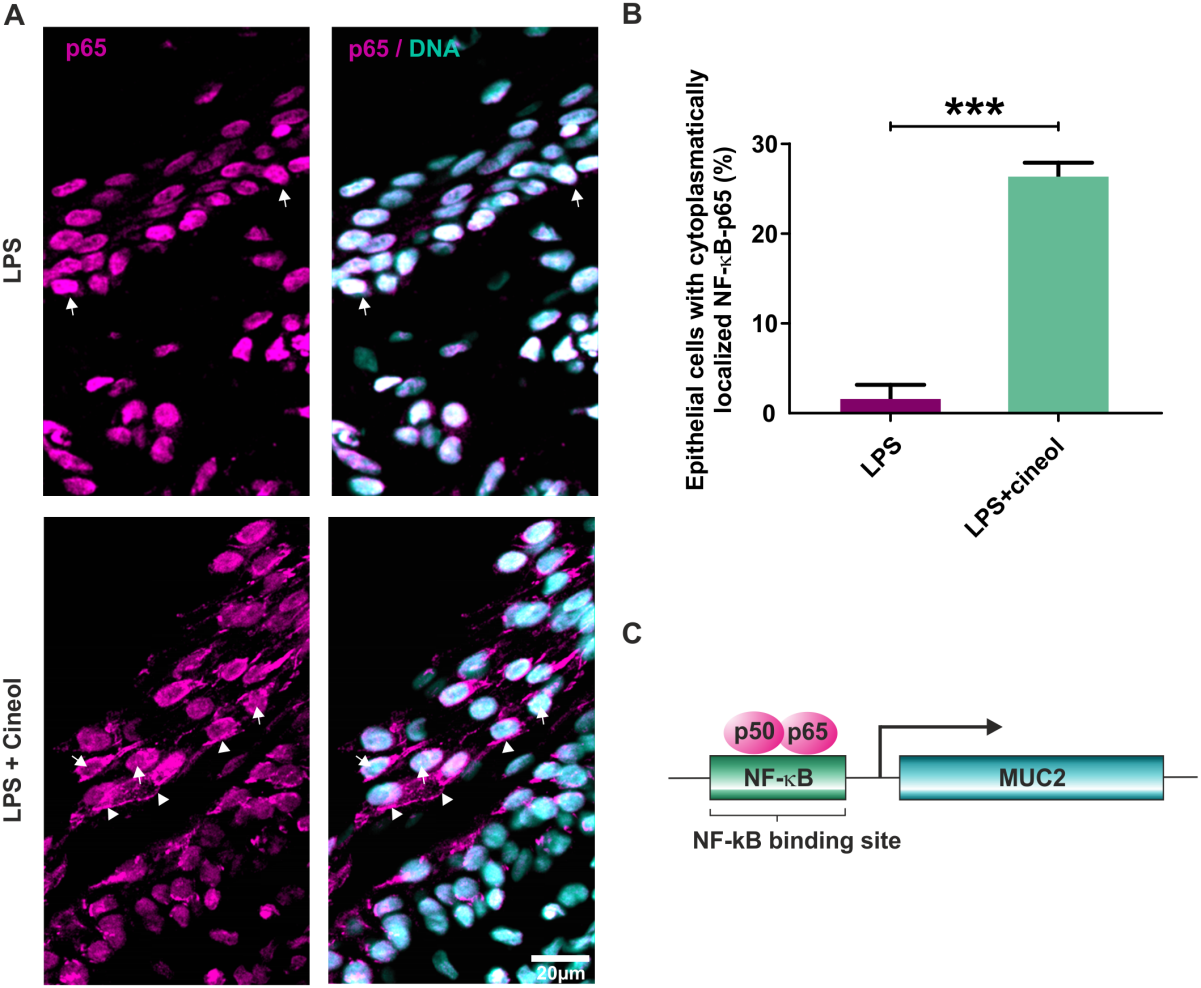
Nasal slice cultures exposed to 1,8-cineol show reduced activity of NF-κB. **A** Immunocytochemistry of LPS-treated nasal slice cultures revealed nucleus localization of NF-κB-p65 (upper panels, arrows). Co-treatment with LPS and 1,8-cineol resulted in reduced amounts of nuclear NF-κB-p65 (lower panels, arrows) and localization of NF-κB-p65 in the cytoplasm (lower panels, arrowheads). Scale bar: 20μm. **B**: Quantification of immunocytochemical analysis showed significantly increased numbers of epithelial cells with cytoplasmic NF-κB-p65 after LPS and 1,8-cineol co-treatment in comparison to the LPS-approach, indicating a significantly reduced NF-κB-activity. ***p < 0.001 was considered significant (t-test). **C**: Schematic view of NF-κB activating MUC2 gene expression via binding to a κB-binding site in 5’ region of the MUC2 gene (31).

The transcription factor NF-κB is an essential regulatory factor of MUC gene-expression [29, 30]. Therefore, its activity was determined in *ex vivo* cultured nasal slices exposed to LPS. Nasal slice cultures co-treated with LPS and 1,8-cineol exhibited significantly reduced expression levels of the NF-κB-target gene TNFα in comparison to its LPS-dependent stimulation (Fig 3D). Immunocytochemistry of LPS-treated nasal slice cultures revealed localization of NF-κB-p65 within the nucleus, further indicating NF-κB-activity (Fig 4A upper panels, arrows). Co-treatment with LPS and 1,8-cineol resulted in reduced amounts of nuclear NF-κB-p65 (Fig 4A, lower panels, arrows) and localization of NF-κB-p65 in the cytoplasm (Fig 4A, lower panels, arrowheads). Quantification of immunocytochemical analysis showed significantly increased numbers of epithelial cells with cytoplasmic NF-κB-p65 in response to 1,8-cineol co-treatment in comparison to the LPS-approach (Fig 4B). The significantly reduced NF-κB-activity is suggested to result in the observed decrease in MUC2-gene expression levels (Fig 4C)[31].

## Discussion

This study shows for the first time a reduction of mucus-production in *ex vivo* cultured human nasal slices after 1,8-cineol treatment during experimental rhinosinusitis. Overproduction of mucins is commonly associated with inflammatory airway diseases, such as rhinosinusitis (8) and COPD ([2, 3]. In this study, we used LPS to mimic bacterial superinfection common during late rhinosinusitis [28]. Exposure of *ex vivo* cultivated nasal slices to LPS resulted in significantly increased density of mucin-filled goblet cells, reflecting the pathological situation during rhinosinusitis [8]. In agreement with our findings, an increased mucin gene expression was observed occurring in an LPS-dependent manner during the pathogenesis of cystic fibrosis lung disease [30].

LPS was also described to be applicable for studying effects of 1,8-cineol during inflammation within several distinct cellular and animal models [24, 32, 33]. In our study, co-incubation of nasal slice cultures with LPS and 1,8-cineol led to a significantly decreased number of mucin-filled goblet cells. These observations further highlight the previously suggested role of 1,8-cineol in controlling mucus hypersecretion by cytokine inhibition [32]. In accordance to our findings, Bastos and colleagues likewise reported in 2011 indications for the prevention of mucus accumulation in OVA-challenged guinea pigs by 1,8-cineol [34]. Emphasizing the clinical relevance of the presented data, Kehrl and coworkers demonstrated the successful application of 1,8-cineol for treating rhinosinusitis in a randomized, double blind, placebo-controlled trial with 152 patients [18].

Similar to physiological conditions, we found LPS-stimulated gene expression levels of MUC2 and MUC19 to be significantly decreased after 1,8-cineol-treatment. Although MUC5AC and MUC5B are also mainly involved in mucus production [5], MUC2 expression is strongly associated with inflammatory airway diseases [11, 12, 30]. MUC2 was described to be inducible by TNFα in human airway epithelial cells [35] as well as by LPS in a colon epithelial cell line [30], bronchial explants and airway epithelial cells [23]. MUC2 expression is exclusively found in goblet cells [8] and regulated by the transcription factor NF-κB [29, 30], which activates MUC2 gene expression by binding to a κB-binding site in 5’ region of the MUC2 gene [31] (Fig 4). Recently, our group demonstrated that 1,8-cineol strongly inhibits NF-κB-activity by reducing nuclear translocation of NF-κB-p65, which in turn resulted in strongly attenuated expression of pro-inflammatory NF-κB target genes [24]. In particular, 1,8-cineol significantly reduced the expression of TNFα [24], the major regulator of rhinosinusitis disease mediation [21]. Using a mouse model of LPS-induced acute lung injury, Zhao and colleagues validated the NF-κB-associated reduction of inflammation after 1,8-cineol-treatment [33]. Accordingly, 1,8-cineol also led to a reduced NF-κB-activity in *ex vivo* cultivated nasal slices in the present study, suggesting the observed reduced mucus-production and gene expression of MUC2 occurs in an NF-κB-dependent manner.

Kerschner and coworkers demonstrated upregulated expression levels of MUC19 in response to pro-inflammatory cytokines such as tumor necrosis factor alpha (TNFα) in the middle ear epithelium [36]. The present study further extends these findings by showing for the first time an increased MUC19-expression within a model system of bacteria-induced rhinosinusitis. Similar to MUC2, we show reduced MUC19 expression after 1,8-cineol-treatment in association with significantly attenuated NF-κB-activity.

**Fig 5:**
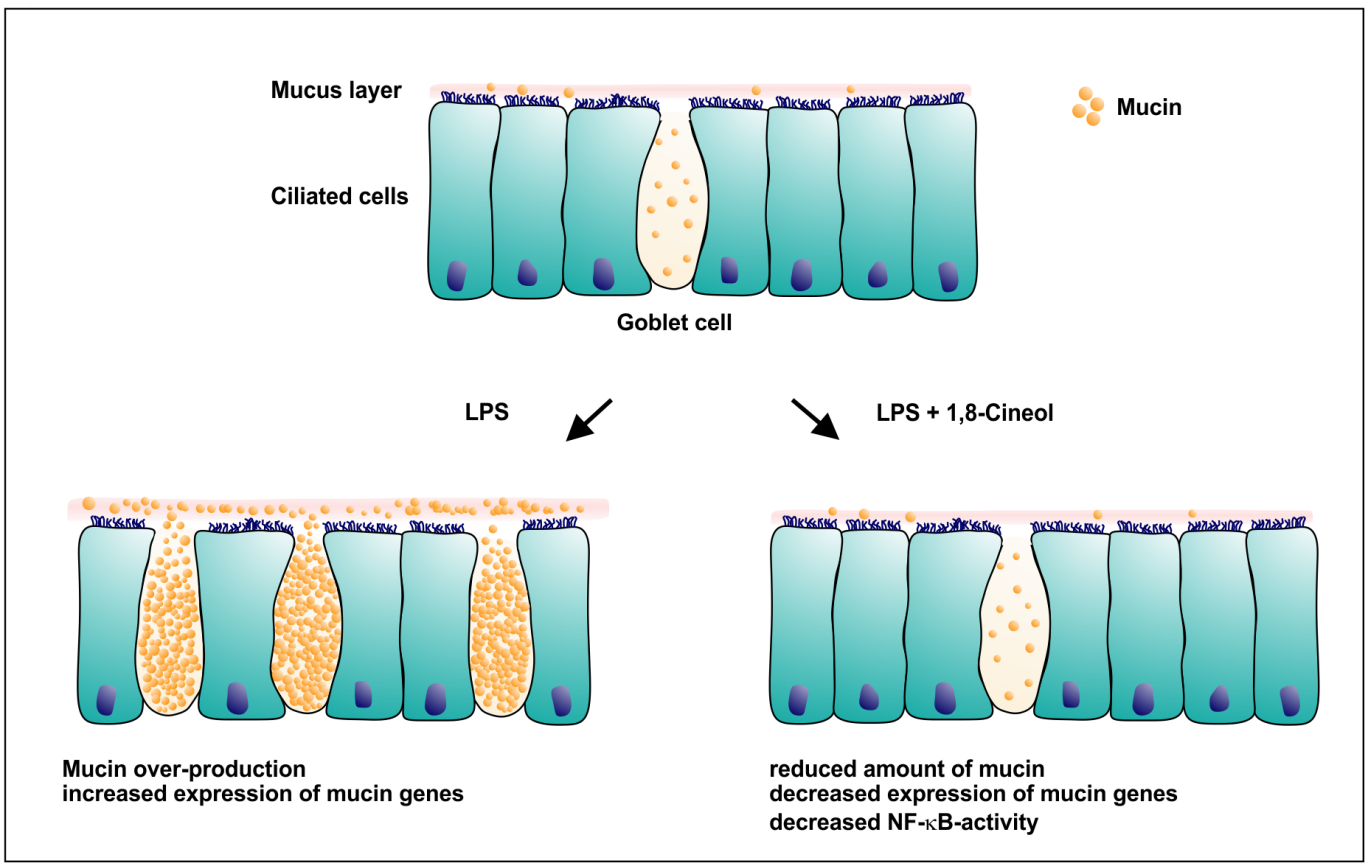
Schematic view on nasal epithelium containing mucus-filled goblet cells during LPS-induced rhinosinusitis and after co-treatment with 1,8-cineol. Co-treatment with LPS and 1,8-cineol leads to reduced production of mucin and decreased expression levels of mucin genes closely associated with attenuated NF-κB-activity.

Here, we could demonstrate a 1,8-cineol-dependent reduction of mucus-production and particularly MUC19 and MUC2 gene expression in *ex vivo* cultured human nasal slices in a novel model for experimental rhinosinusitis (Fig 5). Given the NF-κB-dependent regulation of MUC2 gene expression [31] and the observed reduction of NF-κB-activity, the effects of 1,8-cineol on mucus production and MUC2/MUC19 gene expression likely occur in a NF-κB-dependent manner.

## Conclusion

Treatment of rhinosinusitis, COPD or bronchial asthma strongly requires the reduction of mucus overproduction, a hallmark of inflammatory airway diseases. For the first time our findings demonstrate a 1,8-cineol-dependent reduction of mucus-production and expression of the NF-κB target gene MUC2 which was associated with a reduced NF-κB-activity in *ex vivo* cultured human nasal slices. This system represents a novel model for experimental rhinosinusitis. The beneficial effects of 1,8-cineol on mucin overproduction presented here suggest novel therapeutic medical approaches and may broaden the fields of application of 1,8-cineol. Therefore, topical application of 1,8-cineol may offer a therapeutic approach to reduce bacteria-induced mucus hypersecretion.

## Supporting Information

S1 Fig 1,8-cineol-treatment does not affect viability of the nasal slice cultures. In comparison to inferior turbinate tissue (upper panels), cultured nasal slices showed unchanged low amounts of apoptotic Caspase 3-expression cells (lower panels). Treatment of nasal slice cultures with LPS as well as co-treatment with LPS and 10^-4^M 1,8-cineol did not result in increased amounts of Caspase 3-expressing cells compared to untreated control.
(TIF)
