# Supplementary figures and images for: 1,8-Cineol Reduces Mucus-Production in a Novel Human Ex Vivo Model of Late Rhinosinusitis
Source: PLoS One. 2015 Jul 24;10(7):e0133040. doi: 10.1371/journal.pone.0133040 (PMC4514714; doi:10.1371/journal.pone.0133040)

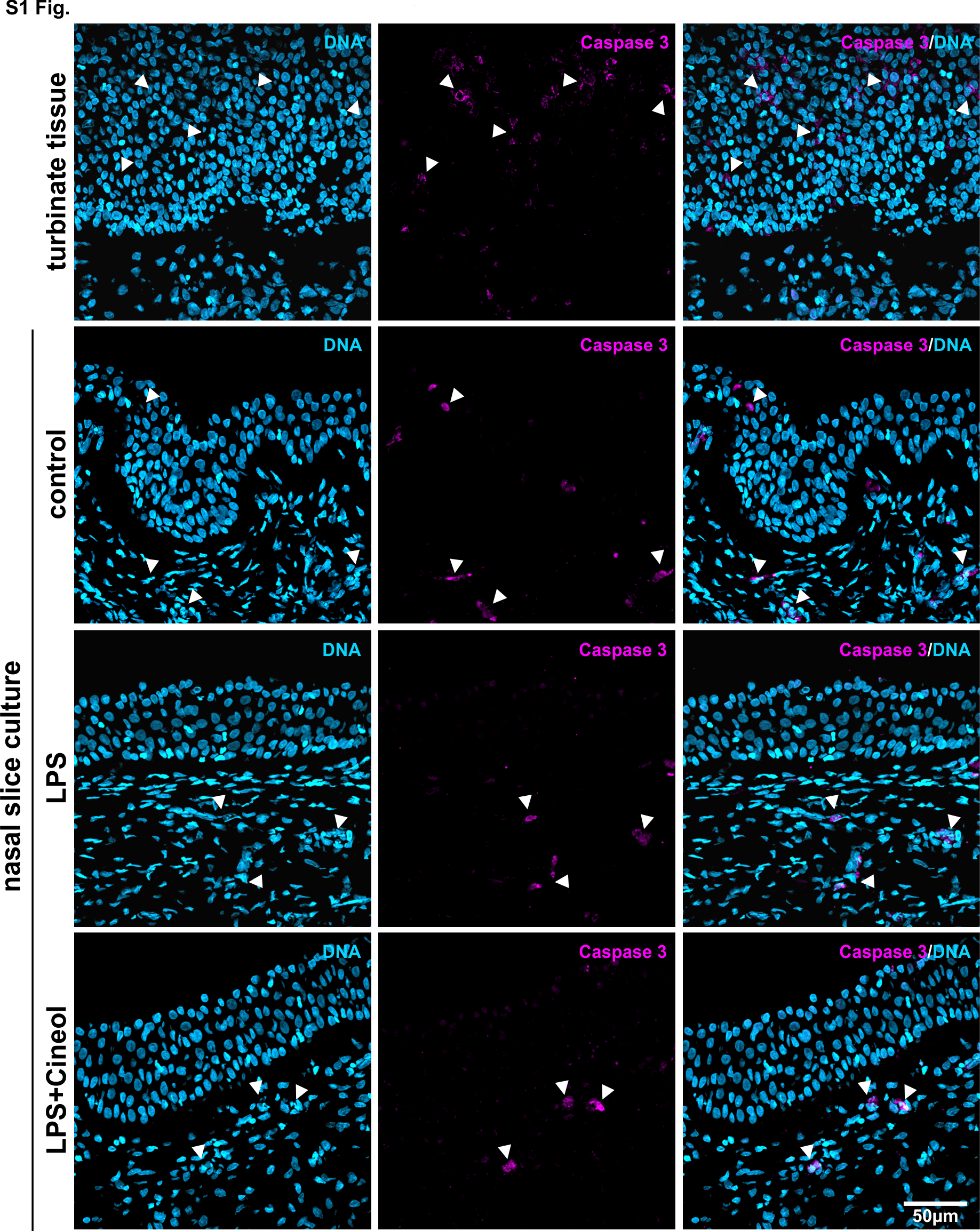

Supplement: S1 Fig — In comparison to inferior turbinate tissue (upper panels), cultured nasal slices showed unchanged low amounts of apoptotic Caspase 3-expression cells (lower panels). Treatment of nasal slice cultures with LPS as well as co-treatment with LPS and 10-4M 1,8-cineol did not result in increased amounts of Caspase 3-expressing cells compared to untreated control. (TIF) [file pone.0133040.s001.tif]
